# Identification of ground-state spin ordering in antiferromagnetic transition metal oxides using the Ising model and a genetic algorithm

**DOI:** 10.1080/14686996.2017.1300046

**Published:** 2017-03-28

**Authors:** Kyuhyun Lee, Yong Youn, Seungwu Han

**Affiliations:** ^a^ Department of Materials Science and Engineering and Research Institute of Advanced Materials, Seoul National University, Seoul, Republic of Korea

**Keywords:** Antiferromagnetic spin ordering, first-principles calculations, genetic algorithm, Ising model, 60 New topics/Others, 401 1st principle calculations

## Abstract

We identify ground-state collinear spin ordering in various antiferromagnetic transition metal oxides by constructing the Ising model from first-principles results and applying a genetic algorithm to find its minimum energy state. The present method can correctly reproduce the ground state of well-known antiferromagnetic oxides such as NiO, Fe_2_O_3_, Cr_2_O_3_ and MnO_2_. Furthermore, we identify the ground-state spin ordering in more complicated materials such as Mn_3_O_4_ and CoCr_2_O_4_.

## Graphical abstract

**Figure:**
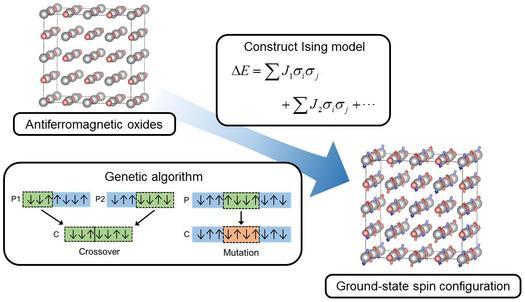

## 1. Introduction

Transition metal oxides (TMOs) are important topics in many studies due to their rich physics [1]. They are also key functional materials in numerous energy and electronic devices, including Li-ion batteries [2–5], photoelectrochemical cells [6,7], catalysts [8–13], and resistance switching memory [14–16]. The structural diversity and variety in the *d*-electron configuration enable diverse functionalities of TMOs, leading to wide applications.

Due to the localized *d* electrons, transition metal atoms exhibit large local magnetic moments, and most TMOs show magnetic ordering such as ferro-, ferri-, and antiferro-magnetism. In particular, TMOs are usually antiferro- or ferri-magnetic materials due to the superexchange coupling between local moments of cations that is mediated by oxygen *p* orbitals. Unlike ferromagnetic materials, there are many degrees of freedom in how spins are ordered in antiferromagnetic TMOs. Experimentally, the spin ordering is directly revealed by neutron diffraction [17–21]. However, it requires strenuous efforts, and only a small number of antiferromagnetic oxides are known for their spin configurations.

The dearth of information on the spin configuration poses a serious problem in the first-principles calculations on TMOs because the method requires specific information on the spin ordering. In theoretical studies of antiferromagnetic oxides, therefore, ground spin configurations are usually found by comparing the energy between a limited set of spin configurations that are chosen rather intuitively. For oxides with simple magnetic ordering such as NiO [22], Fe_2_O_3_ [23], LaTiO_3_[24] and Mn_3_O_4_ [25], the spin alignment in the ground state was correctly identified in this way. However, for complicated TMOs that include several different species of magnetic ions, the number of possible spin distributions increases exponentially and it would be difficult to choose candidate configurations intuitively.

In this article, we propose a general method for finding the most stable spin configuration of magnetic materials by combining the first-principles calculations, the Ising model, and a genetic algorithm. For the Ising model, atom pairs are classified depending on the pair distance and the number of shared oxygen atoms. The most stable magnetic ordering is found through the genetic algorithm applied on the Ising model. To validate the method, we investigate the ground-state magnetic ordering of various TMOs such as NiO, Fe_2_O_3_, Cr_2_O_3_, MnO_2_, Mn_3_O_4_, and CoCr_2_O_4_, and compare the results with previous studies.

## 2. Methods

**Figure 1.**
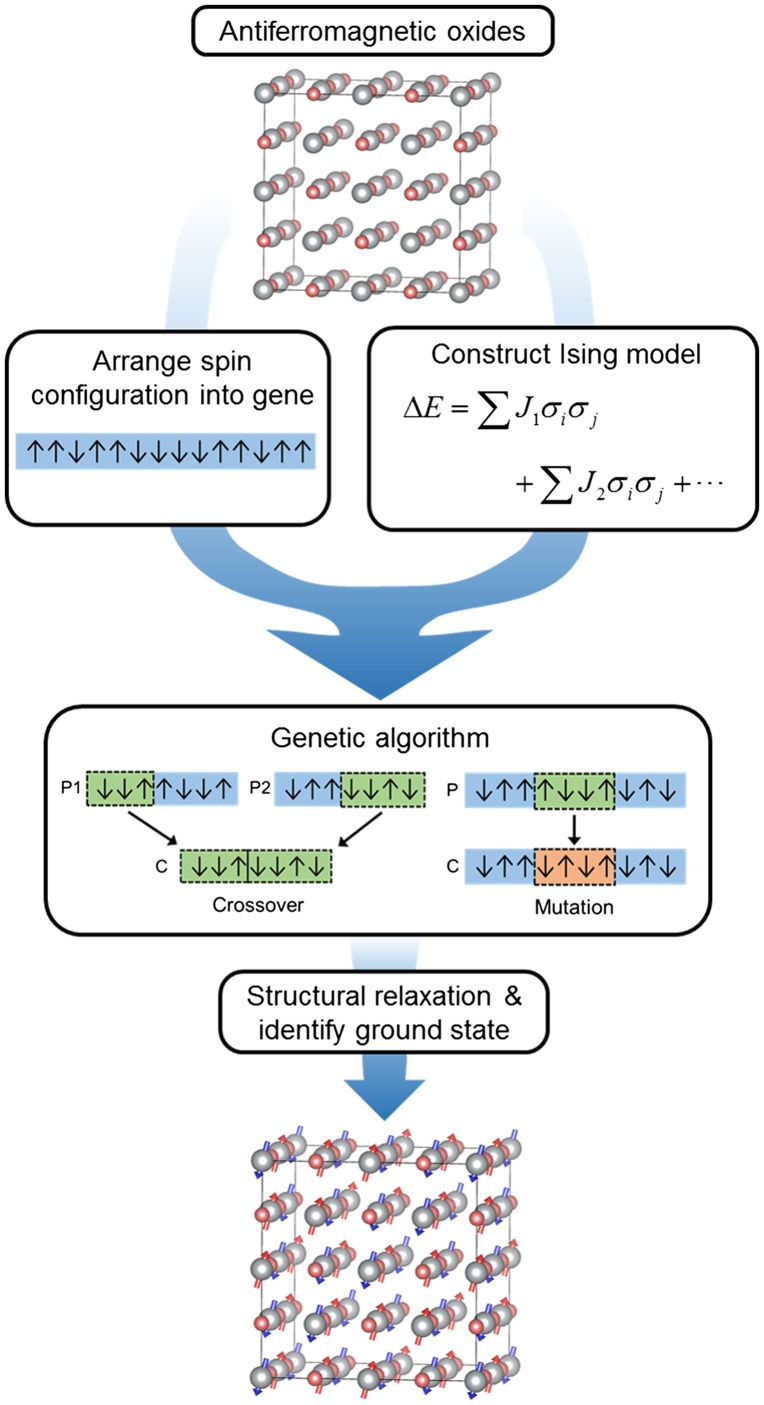
Schematic diagram for finding ground-state magnetic ordering in the antiferro-magnetic materials.

Figure 1 presents the overall computational procedure used in this work. Briefly, we first parameterize magnetic interactions in a certain TMO within the Ising model on the basis of first-principles results on a specific set of spin configurations. To obtain the minimum energy state in the Ising model, we take a large supercell and encode the spin configuration within the supercell into a one-dimensional gene and apply the genetic algorithm. As a result, candidate spin configurations are obtained, for which we carry out the first-principles calculations with full structural relaxations and identify the final ground state. The details in each step are discussed in the following subsections.

### 2.1. Parameterization of spin–spin interactions

Our primary assumption is that the magnetic exchange energy is well described by the Ising model. This limits the application scope of the present method to the collinear magnetic systems. In the Ising model, the exchange energy is expressed as a sum of interactions between spin pairs:

(1) $$\Delta E=\sum_{i<j}J_{ij}\sigma_{i}\sigma_{j}$$

where *i* and *j* indicate the atomic sites with finite spin moments, and *σ* and *J* indicate the magnetic moment of each atom and the exchange energy between cation pairs, respectively. In parameterizing {*J*_*ij*_}, we assume that the magnitudes of local spin moments are constant regardless of spin ordering. This means that *σ*_*i*_ in Equation (1) takes only +1 or –1. We confirmed that for the materials studied in the present work, the variation of local magnetic moments is always less than 5%. We classify cation pairs according to the pair distance and number of shared oxygen atoms, and assign independent *J*_*ij*_ for each type of cation pairs. As the distance between the atomic pairs becomes longer, the overlap of the electron density and the strength of magnetic interaction decreases. From several tests, we confirmed that the spin–spin interactions are negligible if the pair distance is larger than twice the longest first-neighbor bond length, which defines the cutoff range of *J*_*ij*_. In this way, the Ising model for NiO in the rock-salt structure is expressed by two *J*_*ij*_ parameters, for instance.

**Figure 2.**
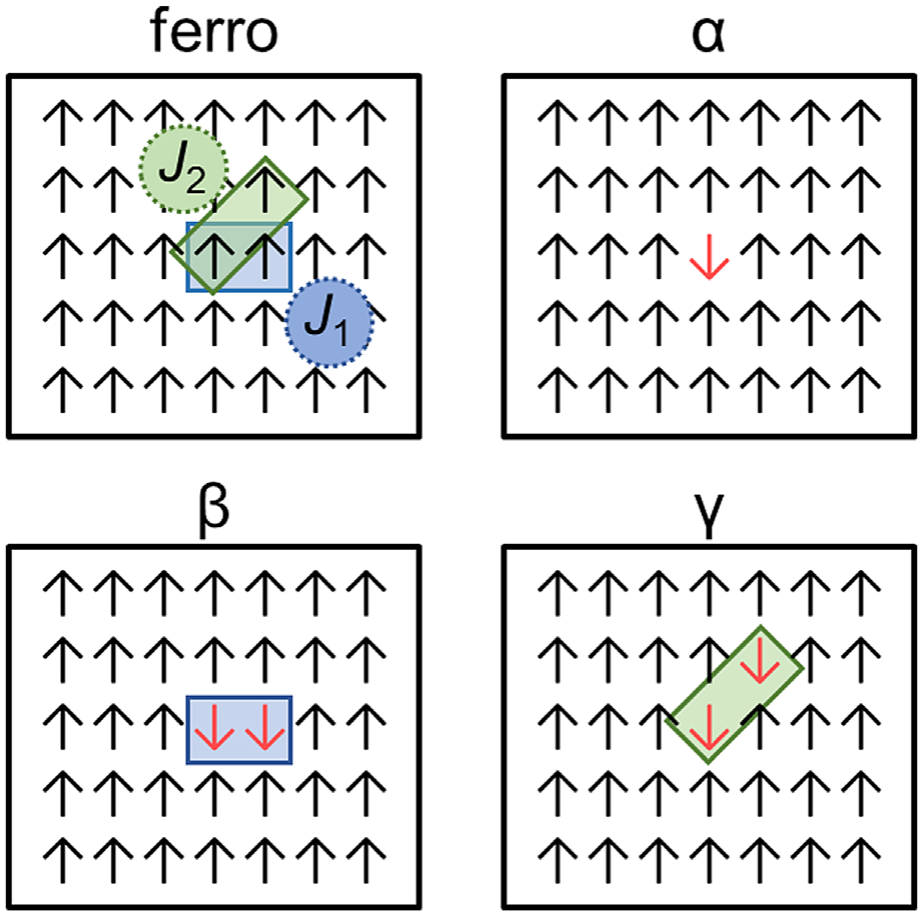
Model spin system in the square lattice. There are two exchange parameters, *J*_1_ and *J*_2_, for the nearest and second-nearest pairs, respectively. The four spin configurations (ferro, *α*, *β*, and *γ*) are used in evaluating *J*_1_ and *J*_2_.

Each *J*_*ij*_ can be evaluated from the difference in the first-principles energy between spin configurations. We depict the procedure using the simplified two-dimensional spin lattice as shown in Figure 2. Suppose that there are two independent exchange interactions, *J*_1_ and *J*_2_, as marked in the ferromagnetic configuration, which correspond to the nearest and next-nearest neighbor interactions, respectively. We carry out first-principles calculations on the four spin configurations in Figure 2 and obtain the total energy for each configuration. It is straightforward to show that *J*_1_ and *J*_2_ are obtained from the Ising model as follows:

(2) $$J_{1}=\frac{1}{4}\left(E_{\text{ferro}}+E_{\beta }-2E_{\alpha }\right)$$

(3) $$J_{2}=\frac{1}{4}\left(E_{\text{ferro}}+E_{\gamma }-2E_{\alpha }\right)$$

where *E*_*i*_ is the first-principles energy for the configuration *i*. This can be extended to the general cases: the exchange parameter for any spin pair *ij* can be obtained from the energies of four spin configurations:

(4) $$J_{ij}=\frac{1}{4}\left(E_{\text{ferro}}+E_{ij}-E_{i}-E_{j}\right)$$

where *E*_*ij*_ is the energy of the structure in which the spins of atom *i* and *j* are flipped from the ferromagnetic configuration while *E*_*i*(*j*)_ is the energy with only spin *i*(*j*) reversed. In choosing a computational cell for the first-principles calculations, we expand the unit cell such that the magnetic interactions between periodic images become negligible. The structural relaxation is not considered here.

### 2.2. Finding the ground state using the genetic algorithm

**Figure 3.**
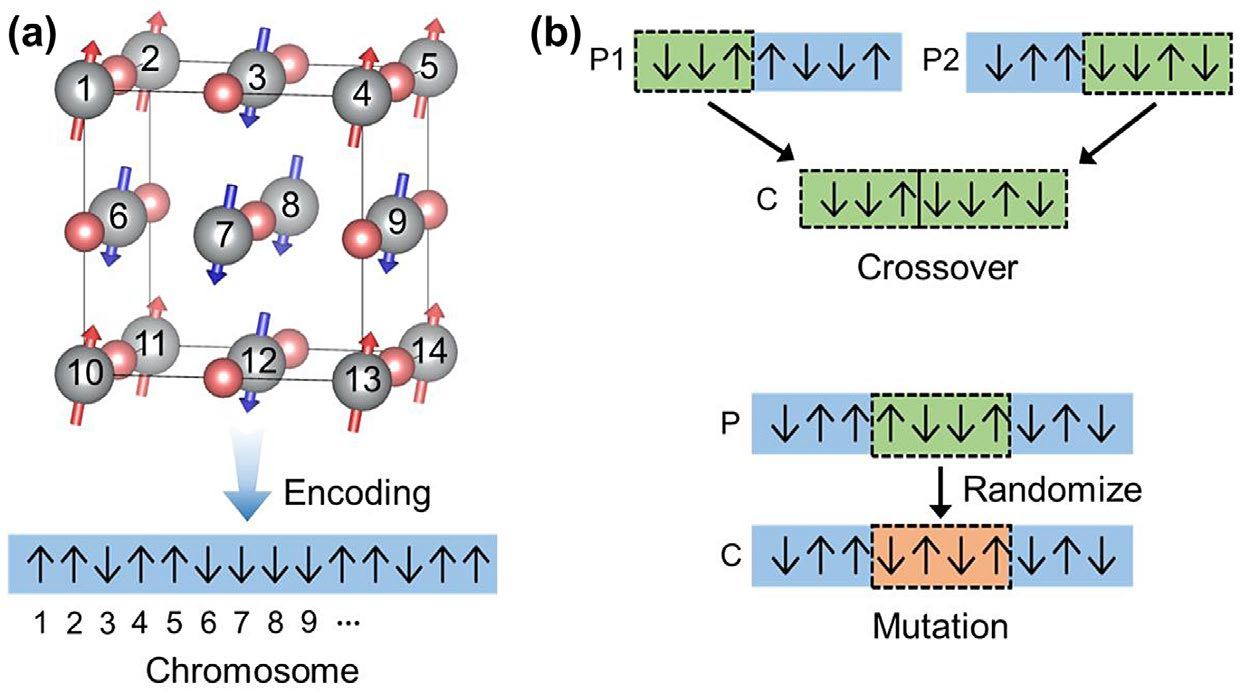
(a) Schematic diagram for generating a chromosome by encoding spin directions. (b) Schematic diagram of genetic operators, crossover and mutation. P and C mean the parent and child, respectively.

The genetic algorithm is used to find the most stable magnetic spin ordering from the constructed Ising model. We use a supercell that is large enough to describe various antiferromagnetic configurations. Figure 3(a) shows an example of encoding the spin configuration into a chromosome. Each gene corresponds to the spin direction (up or down) of a magnetic atom. For the genetic operators, we employ crossover and mutation as shown in Figure 3(b). The crossover operator splits two chromosomes (P1 and P2) in the same manner and combines different regions to generate the child chromosome (C). In the mutation, the genes in a selected region are randomized. We determine the length of mutation region in proportion to the chromosome size.

In the first generation, chromosomes are randomly generated. The population in a generation is fixed to 64 and we iterate the generational process 500 times. In each generation, parents are selected by the possibility *p*_*i*_ following the roulette-wheel selection:

(5) $$p_{i}=\frac{f\left(E_{i}\right)}{\sum_{k=1}^{N}f\left(E_{k}\right)}$$

where *i* and *N* indicate the index of chromosome and the total population, respectively. In Equation (5), *f*(*E*_*i*_) is the fitness function with the following form:

(6) $$f\left(E_{i}\right)=E_{\max}-E_{i}+\frac{3}{2}\left(E_{\max}-E_{\min}\right)$$

where *E*_max_ and *E*_min_ are the maximum and minimum energies in each generation, respectively. The new population is generated through genetic operators (16 for crossover, 16 for mutation). In addition, we introduce 16 random genes in every generation to escape from the local minimum and retain the 16 lowest-energy configurations from the previous generation.

### 2.3. First-principles calculations for candidate configurations

As a final step, we choose the five lowest energy structures in the last generation of genetic algorithm. Then, we carry out the full structural relaxations using first-principles calculation and identify the most stable spin configuration.

### 2.4. Computational programs

We use VASP (Vienna Ab-initio Simulation Package) to perform first-principles calculations [26] and in-house code for the genetic algorithm. We use the projector-augmented wave method [27] within the generalized gradient approximation (GGA) [28]. The on-site Coulomb repulsion term (*U*) is also applied. The *U* values for each transition metal are chosen from the previous work [29]. A cutoff energy of 500 eV is used for all materials and **k**-point convergences are carefully tested. The initial structures are taken from the Inorganic Crystal Structure Database [30].

## 3. Results and discussion

**Figure 4.**
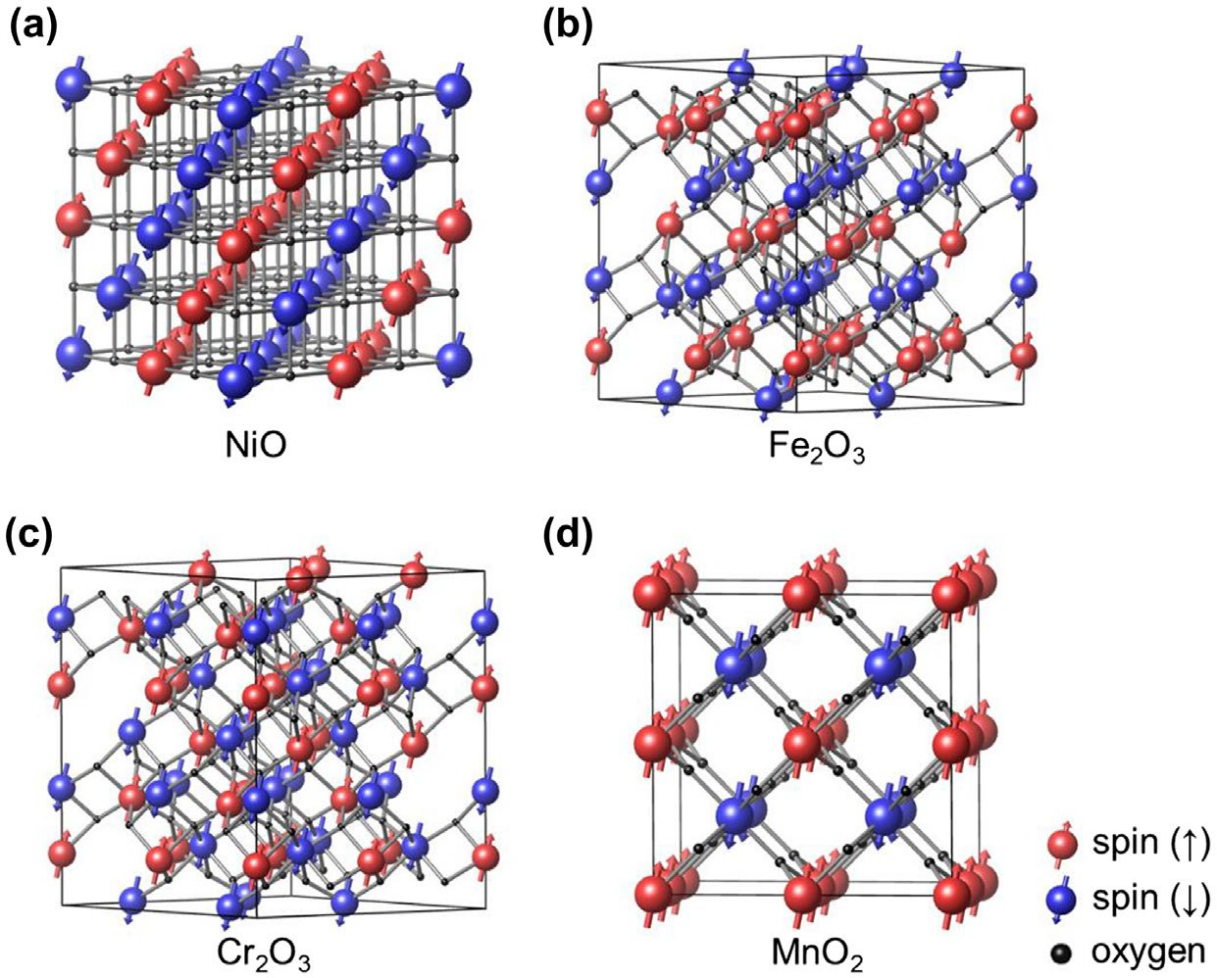
The most stable magnetic ordering of (a) NiO, (b) Fe_2_O_3_, (c) Cr_2_O_3_ and (d) MnO_2_. The cell boundary indicated by black lines corresponds to the unit supercell used for applying the genetic algorithm. The ground-state spin arrangements of metal ions obtained by the present method are indicated by red (spin-up) and blue (spin-down) spheres.

In order to validate the present approach, we first try to find the ground-state spin configuration of oxides such as NiO (rocksalt), Fe_2_O_3_ (*α* phase), Cr_2_O_3_ (corundum) and MnO_2_ (*β* phase) whose antiferromagnetic orderings are well established by experiments [17,18,31,32]. Figure 4 shows the supercell of each material used in calculation. The computed {*J*_*i*_} are presented in Table 1.

**Table 1.** Exchange interaction parameters of NiO, Fe_2_O_3_, Cr_2_O_3_, and MnO_2_.

Material	*J*_1_	*J*_2_	*J*_3_	*J*_4_	*J*_5_
NiO	–0.9(2.95)	14.4(4.17)			
Fe_2_O_3_	3.0(2.89)	3.6(2.97)	40.1(3.36)	28.0(3.70)	1.9(3.98)
Cr_2_O_3_	15.9(2.65)	11.7(2.89)	–4.6(3.43)	–5.4(3.65)	
MnO_2_	4.0(2.88)	4.4(3.43)			

Notes:

The units are meV. The numbers in parenthesis are the distances between magnetic ions in Å.

**Figure 5.**
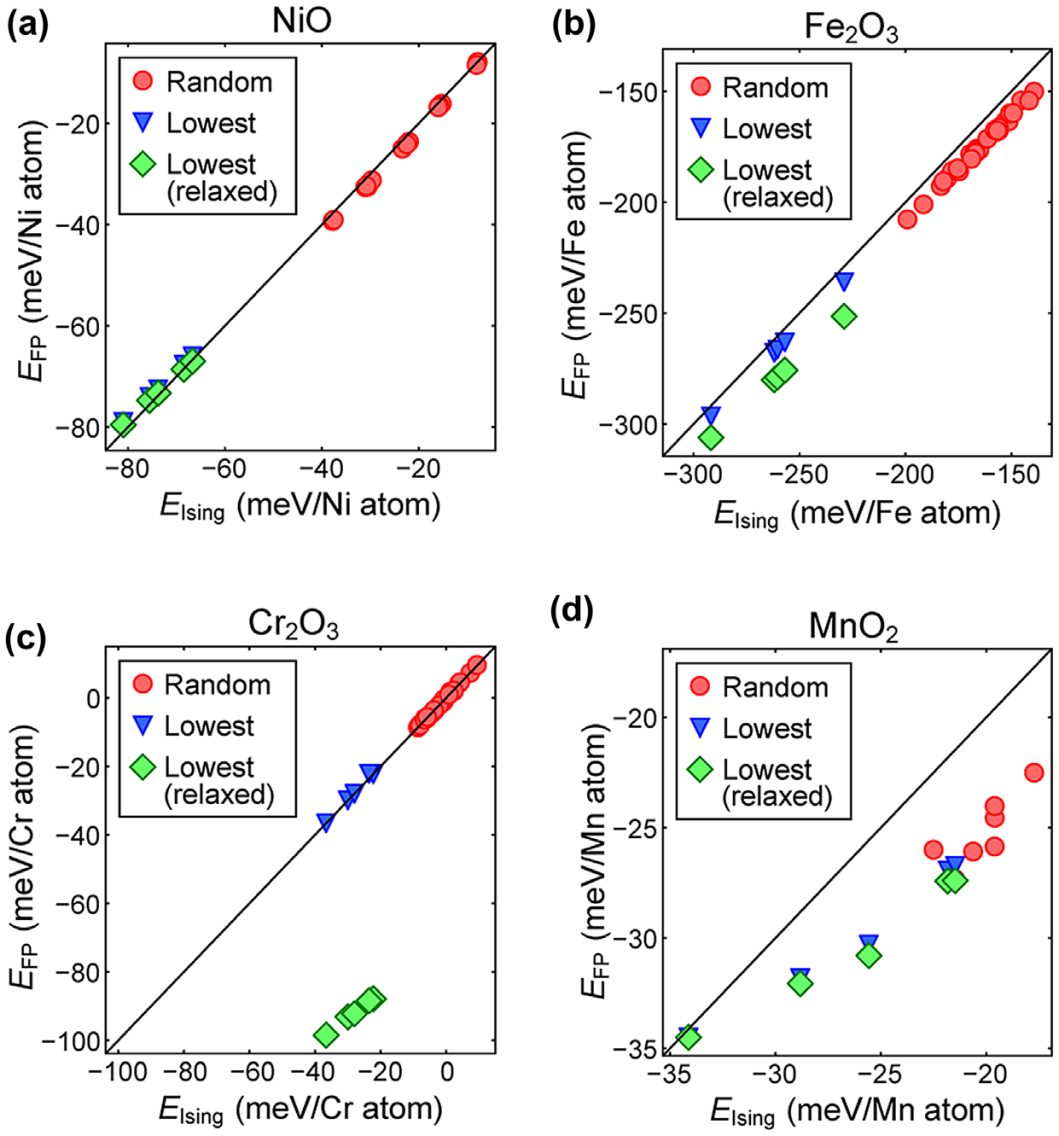
The energy from the Ising model (*E*_Ising_) versus the corresponding energy from first-principles calculations (*E*_FP_) for (a) NiO, (b) Fe_2_O_3_, (c) Cr_2_O_3_, and (d) MnO_2_. The energy is referenced to the ferromagnetic spin orientations. Red circles indicate the test sets with random spin orientations. Blue triangles indicate five candidates from the genetic algorithm and green diamonds are their relaxed energies from the first-principles calculations.

To verify the constructed Ising model, we compare in Figure 5 the energy of various spin configurations between the Ising model (*x* axis) and first-principles calculations (*y* axis), shown as solid circles. The spin orientations in the test sets are generated randomly under the constraint that the total spin moment is zero (Σ_*i*_ *σ*_*i*_ = 0). In Figure 5, the energy zero corresponds to the ferromagnetic spin orientations in which all *σ*_*i*_ in Equation (1) are 1. It is seen that the energies from the Ising model agree well with first-principles results, confirming the validity of the constructed Ising model. In the case of MnO_2_, the exchange interactions in Table 1 are much weaker than for other materials, resulting in small energy differences among spin orientations.

**Figure 6.**
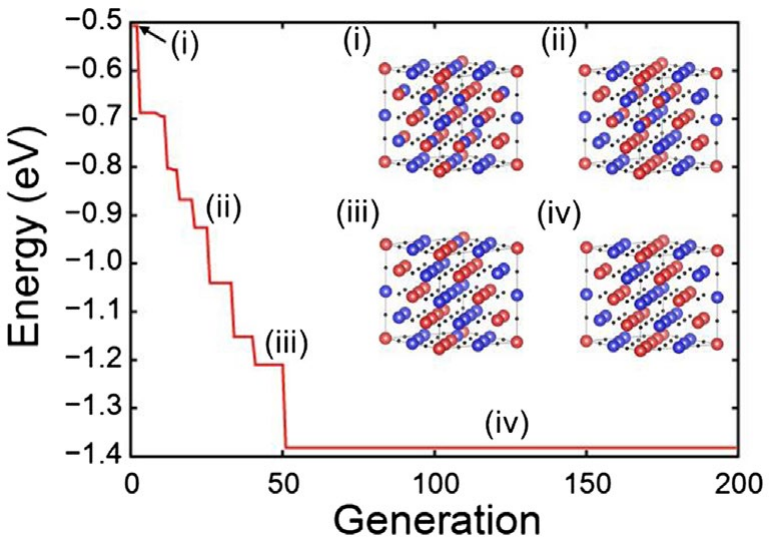
The lowest magnetic energy for NiO in each generation of genetic algorithm. Inset figures show the most stable spin arrangements. The red (blue) spheres indicate spin up (down) Ni atoms.

As an example of the genetic procedure, the results for NiO are displayed in Figure 6. The lowest energy for each generation in the genetic algorithm is shown and the most stable spin configurations at certain generations are drawn as insets. The final spin configuration (iv) corresponds to the type-II antiferromagnetic ordering, which is consistent with experiments [17] as well as previous theory [22].

We identify the five lowest energy configurations through the genetic algorithm as indicated by blue triangles in Figure 5. The lengths of genes are 32, 48, 48 and 16 for NiO, Fe_2_O_3_, Cr_2_O_3_ and MnO_2_, respectively. The agreements with the first-principles calculations are still good for the candidate orderings, implying that the Ising model works throughout the whole energy range. Finally, the candidate structures are fully relaxed (both ionic positions and lattice parameters) within the first-principles method. The relaxed energies are indicated by the green diamonds in Figure 5. The overall rigid shifts between triangles and diamonds mean that the relaxation energies are similar among the structures. Except for Cr_2_O_3_, the relaxations of ions and lattice parameters are very small, and the magnitude of relaxation energy is much smaller than for the exchange energy. For Cr_2_O_3_, we find that the lattice expands substantially (~0.1 Å) and it is similar among the spin configurations.

The final ground-state spin configurations of NiO, Fe_2_O_3_ and Cr_2_O_3_ are presented in Figure 4. They are consistent with experimental data as well as previous theoretical results [17,18,31]. We note that helical spin order was found for MnO_2_ in an experiment [32], while the present model is limited to the collinear magnetization. Expanding the current model to the non-collinear magnetism, possibly on the basis of Heisenberg model, would constitute future work. Nevertheless, the present result agrees well with the other theoretical results assuming collinear magnetism [33].

**Figure 7.**
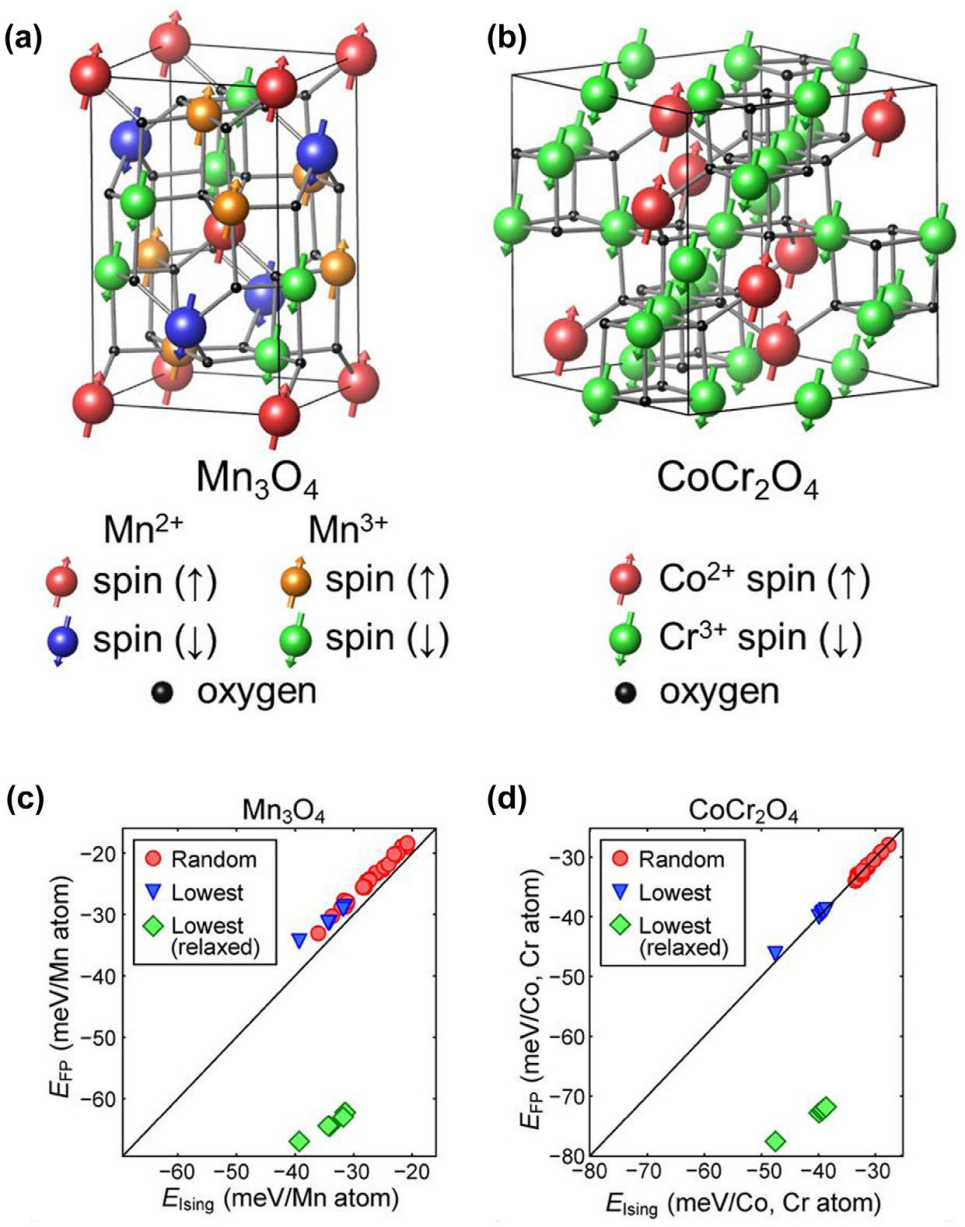
The most stable spin structure of (a) Mn_3_O_4_ and (b) CoCr_2_O_4_ from the genetic algorithm. In (a), red (blue) spheres mean spin up (down) of Mn^2+^ ions and orange (green) spheres indicate spin up (down) of Mn^3+^ ions. In (b), red (green) spheres correspond to the spin-up Co^2+^ (spin-down Cr^3+^). (c) and (d) compare the magnetic energies between Ising model and first-principles calculations for Mn_3_O_4_ and CoCr_2_O_4_, respectively. The notations are the same as in Figure 5.

We apply the present approach to more complex systems that include several types of magnetic atoms. We choose Mn_3_O_4_ (spinel) and CoCr_2_O_4_ (spinel). In Mn_3_O_4_, there are two types of valence for Mn, Mn^2+^ and Mn^3+^, while both Co and Cr atoms possess local spin moments in CoCr_2_O_4_. Since the magnitude of magnetic moments depends on the valence state, the multivalent system such as Mn_3_O_4_ should be treated as if there are two types of magnetic atoms. The supercell is described in Figure 7(a) and 7(b), the computed {*J*_*i*_} are presented in Table 2. Although the magnetic structures are more complicated, the agreements between the constructed Ising model and first-principles calculations are comparable to the previous cases, as shown in Figure 7(c) and 7(d).

**Table 2:** Exchange interaction parameters of Mn_3_O_4_ and CoCr_2_O_4_.

Material	*J*_1_	*J*_2_	*J*_3_	*J*_4_	*J*_5_
Mn_3_O_4_	17.4 (2.88)	2.9 (3.12)	–0.9 (3.43)	3.3 (3.73)	7.8 (3.83)
	Mn^3+^-Mn^3+^	Mn^3+^-Mn^3+^	Mn^2+^-Mn^3+^	Mn^2+^-Mn^2+^	Mn^2+^-Mn^3+^
CoCr_2_O_4_	3.3 (2.95)	1.3 (3.36)	5.9 (3.46)		
	Cr^3+^-Cr^3+^	Co^2+^-Co^2+^	Co^2+^-Cr^3+^		

Notes:

The units are meV. The numbers in parenthesis are the distance between magnetic ions in Å. The type of metal ions also noted.

The most stable spin configurations obtained from the genetic algorithm are depicted in Figure 7(a) and 7(b). In the process, we use genes with the length of 12 and 24 for Mn_3_O_4_ and CoCr_2_O_4_, respectively. In Mn_3_O_4_, there are several ground-state spin configurations with the identical energy within the Ising model. The degeneracy is slightly lifted in the first-principles calculation by ~1 meV/Mn atom. The ground-state spin configuration for Mn_3_O_4_ in Figure 7(a) is the same as in [25] in which the minimum energy ordering was found by testing a limited set of spin configurations. The most stable magnetic ordering of CoCr_2_O_4_ are shown in Figure 7(b). It is found that every Co and Cr atom has the same spin-up and spin-down moments, respectively, meaning that the material is in fact ferrimagnetic. In the experiment, CoCr_2_O_4_ is known to have non-collinear spin distributions [21] and so the direct comparison with the present result is not feasible. On average, however, Co^2+^ and Cr^3+^ are in spin-up and spin-down states, respectively, which is consistent with the present result.

## 4. Conclusion

In summary, we proposed a general method to find the ground-state collinear spin configuration of antiferromagnetic materials by constructing the Ising model and applying the genetic algorithm. This present method does not rely on human intuition in selecting the candidate spin ordering. We demonstrated the accuracy and efficiency of the method by identifying the lowest spin configurations of several magnetic oxides such as NiO, Fe_2_O_3_, Cr_2_O_3_, MnO_2_, Mn_3_O_4_, and CoCr_2_O_4_. The present method may find a wide use except for non-collinear magnetic systems, particularly when they deviate strongly from the collinear configurations. We believe that the present scheme could be applied to identifying the antiferromagnetic ordering automatically, which will be particularly useful in massive calculations of transition metal oxides. In this respect, we note that the current digital databases such as AFLOW [34] and Materials Project [35] do not consider the antiferromagnetic ordering.

## Disclosure statement

No potential conflict of interest was reported by the authors.

## Funding

This work was supported by the Technology Innovation Program (or Industrial Strategic Technology Development Program [10052925, Atomistic process and device modeling of sub-10nm scale transistors]) funded By the Ministry of Trade, Industry & Energy(MOTIE, Korea). The computations were carried out at the KISTI Supercomputing Center (KSC-2016-C3-0006).
